# Neuropsychiatric prodromes and symptom timings in relation to disease onset and/or flares in SLE: results from the mixed methods international INSPIRE study

**DOI:** 10.1016/j.eclinm.2024.102634

**Published:** 2024-05-20

**Authors:** Melanie Sloan, James A. Bourgeois, Guy Leschziner, Thomas A. Pollak, Mervi Pitkanen, Rupert Harwood, Michael Bosley, Alessandra Bortoluzzi, Laura Andreoli, Wendy Diment, James Brimicombe, Mandeep Ubhi, Colette Barrere, Felix Naughton, Caroline Gordon, David D’Cruz

**Affiliations:** a Department of Public Health and Primary Care Unit, University of Cambridge, UK; b Department of Psychiatry and Behavioral Sciences, University of California, Davis Medical Center, Sacramento, CA, United States; c Department of Neurology, Guy’s and St Thomas’ Hospitals NHS Foundation Trust, London, UK; d Institute of Psychiatry, Psychology and Neuroscience, King’s College London, and South London and Maudsley NHS Foundation Trust, London, UK; e Swansea University Medical School, Wales, UK; f Patient and Public Co-Investigators; g Rheumatology Unit, Department of Medical Sciences, University of Ferrara and Azienda Ospedaliero-Universitaria S. Anna, Ferrara, Italy; h Unit of Rheumatology and Clinical Immunology, ASST Spedali Civili, Italy; i Rheumatology Research Group, Institute of Inflammation and Ageing, College of Medical and Dental Sciences, University of Birmingham, Birmingham, UK; j Behavioural and Implementation Science Group, School of Health Sciences, University of East Anglia, Norwich, UK; k The Louise Coote Lupus Unit, Guy’s and St Thomas’ Hospitals NHS Foundation Trust, London, UK; l Department of Clinical and Experimental Sciences, University of Brescia, Brescia, Italy

**Keywords:** SLE, Lupus, Neuropsychiatric, Prodrome, Nightmares, Mixed methods

## Abstract

**Background:**

Neuropsychiatric symptoms in SLE and other systemic autoimmune rheumatic diseases (SARDs) are challenging to diagnose, attribute and manage. We investigated the timings of onset of a broad range of neuropsychiatric (NP) symptoms in relation to timing of SLE onset. In addition, we explored whether NP symptoms may be a prodrome to SARD onset and to subsequent flares.

**Methods:**

We collected patient reports of the timing of their first episode of 29 NP symptoms relative to SLE non-NP symptom onset. Surveys (n = 676 SLE patients and n = 400 clinicians) and interviews (n = 50 clinicians; and n = 69 SARD patients, including 27 SLE patients) were completed from 2022 to 2023, and analysed using mixed methods.

**Findings:**

The majority of NP symptoms did not first present around the time of SLE onset, contrary to the prevailing view among many rheumatology participants and in the literature. For example, among patients who experienced hallucinations, 54% reported first presentation >1 year after disease onset. Patient interviews also revealed that a range of NP symptoms may be a prodrome to SLE/SARDs onset and later flares, including symptoms not represented in existing classification criteria. Evidence of a possible prodromal syndrome was elicited from those patients who experienced hallucinations. Of these, 61% (SLE) and 34% (other SARDs) reported increasingly disrupted dreaming sleep (usually nightmares) prior to their hallucinations. In-depth interviews revealed that progression of symptoms in flares showed a high degree of inter-patient variation, whilst symptom progression was often similar in individual patient’s recurrent flares.

**Interpretation:**

Neuropsychiatric symptoms can first present at any stage in the SLE disease course. Attributional decisions should evaluate timings of NP symptoms in relation to timing of SLE/SARD symptom onset rather than time of diagnosis due to frequent diagnostic delays. Greater recognition of prodromal/early NP symptoms indicating impending SLE flares (and potentially other SARD flares) could enable quicker flare identification and treatment.

**Funding:**

The Lupus Trust.

**Research in context**

**Evidence before this study**

We first searched PubMed on 8 October 2021 using the terms “Neuropsychiatric” OR “Mental health” AND “SLE” OR “lupus” OR “Rheumatology” for all article types with no language or time parameters set. Additional searches then combined individual neuropsychiatric (NP) terms (for example, “hallucinations”) AND SLE OR “Rheumatology”, and later focused on studies including the term “prodrome” AND “neuropsychiatric” and the individual systemic rheumatic autoimmune diseases (SARDs). PubMed searches for relevant new articles continued throughout the study. The literature indicated frequent under-reporting and misattributions of NP symptoms in SLE and other SARD patients, and clinician-patient discordance in NP symptom attribution. There has been extensive research on NPSLE, in particular by the Systemic Lupus International Collaborating Clinics (SLICC) group, and the Italian Society of Rheumatology Study Group on NPSLE. However, the majority of SLE studies were focussed on a limited range of NP symptoms, largely restricted to a maximum of the 19 neuropsychiatric symptoms detailed in the 1999 American College of Rheumatology (ACR) criteria. There was very little literature on prodromal NP symptoms in SLE and other SARDs compared to other diseases such as Multiple Sclerosis.

**Added value of this study**

The INSPIRE (**I**nvestigating **N**europsychiatric **S**ymptom **P**revalence and **I**mpact in **R**heumatology **P**atient **E**xperiences) study elicited patient priorities and symptoms for inclusion directly from patients as opposed to following existing (largely physician-derived) symptom lists and criteria. This ensured we incorporated a broader range of symptoms than any previous SARD research, and included the symptoms of importance to the patients. We identified a potential NP prodrome, including some previously unexplored symptoms, for some SLE patients both preceding full-spectrum disease onset and in recurrent flares. This could provide earlier indicators of disease flares–and thus quicker intervention–in some patients, before the more typical symptoms develop. An example of a prodromal symptom was evidenced by patient reports of disrupted dreaming sleep, particularly nightmares, preceding hallucinations in some SLE and other SARD patients. In addition, initiating discussions of often stigmatised distressing symptoms with questions about sleep disruption may improve–the currently poor–reporting of these symptoms. Data obtained directly from patients has different strengths and limitations compared to the majority of the literature which is currently dominated by data acquired from clinician assessments and medical documents and provides a complementary perspective for actions to improve care.

**Implications of all the available evidence**

There is evidence of an NP prodrome in some SLE patients in common with other diseases, such as Parkinson’s disease and multiple sclerosis (MS), both in disease onset and subsequent recurrent flares. Clinicians should remain vigilant to NP symptoms arising at any stage of the disease course. Timing of disease symptoms’ onset as opposed to date of confirmed diagnosis should be used in attributional decisions due to frequently long diagnostic delays in SLE and many other SARDs, such as Sjögrens. Patient and clinician collaboration in ascertaining, documenting, and monitoring each individual’s usual progression of flare symptoms may provide an “early warning system” for earlier intervention. The possibility of an NP prodrome in some patients with other SARDs requires further exploration.

## Introduction

Systemic lupus erythematosus (SLE) is a complex autoimmune disease characterized by diverse clinical symptoms and potentially affecting multiple organ systems. When symptoms are caused by the direct effect of the disease on the brain and other elements of the nervous system, this is commonly referred to as neuropsychiatric SLE (NPSLE).^1^ Whilst other SLE organ involvement can often be diagnosed by clinician assessment and/or diagnostic tests such as kidney or skin biopsies, NPSLE is more challenging to diagnose and monitor due to the limited sensitivity of objective testing,^2^ and many symptoms being subjective and frequently under-reported.^3^

NPSLE attribution models^4^^,^^5^ assist clinicians in using specified evidence to evaluate the attributability of each NP symptom to SLE, including timelines of NP symptom onset, and favouring factors (i.e., those supporting direct attributability).^4^ Although the Italian NPSLE attribution algorithm uses time of SLE symptom *onset*,^4^ many other studies and clinicians use time of definitive SLE *diagnosis*,^5^^,^^6^ and some exclude pre-SLE diagnosis NP symptoms as unrelated to SLE.^6^ This includes a paper stating that if “the onset of the psychiatric disorder precedes diagnosis of lupus by more than 2 years, the psychiatric disorder should be considered as independent from the illness”.^6^ This strict time window does not allow for the frequent long diagnostic delays in SLE,^7^ which mean that time of diagnosis cannot be assumed to accurately reflect time of disease onset. These long diagnostic delays also often entail frequent misdiagnoses, with a substantial proportion of initial misattributions being psychological problems and/or primary psychiatric illness.^7^ Whilst some of these misdiagnoses likely reflect the widespread lack of knowledge and limited definitive tests for SLE,^2^ it is plausible that some early SLE neurological and/or psychiatric symptoms may represent a NP prodrome for SLE itself. This may also be relevant to the other systemic autoimmune rheumatic diseases (SARDs).

In medicine, a prodrome can be defined as early sign(s) and/or symptom(s) that may indicate the onset of a disease before more diagnostically specific signs and symptoms develop. Recognition of these prodromes offers the “possibility of changing trajectories”^8^ by earlier intervention. Neuropsychiatric prodromal phases are now increasingly recognized in multiple diseases affecting the CNS, including higher rates of depressive disorders in many diseases in the “preclinical and prodromal”^9^ stages.^10^^,^^11^ For example, multiple sclerosis (MS) patients have 50% more clinician consultations for psychiatric illness in the 5 years preceding their MS diagnosis, thought to reflect early subclinical demyelination that commonly antedates the confirmatory objective findings.^11^ Aside from Govoni’s review suggesting that NP symptoms may be a “heralding manifestation” of SLE in 1/3 cases,^12^ and evidence of changes in brain white matter integrity initiating during the early phases of the SLE course,^13^ literature exploring a potential NP prodrome in SLE or other SARDs remains notably scarce. In addition, as far as we know, no studies to date have explored NP symptoms directly with SLE patients in terms of their usual progression of symptoms in an SLE flare.

A recent review article highlighted key challenges when trying to identify neuropsychiatric prodromal phases, including the lack of specificity of these symptoms.^14^ Identifying NP prodromes and progression of symptoms in SLE (and potentially other SARDs) could facilitate earlier initial validated diagnosis of the disease and earlier treatment of recurrent flares, particularly if two main criteria can be demonstrated: 1) within-person similarities of recurrent flare prodrome presentations, and 2) a degree of commonality across patients (i.e., that certain NP symptoms tend to be experienced among many SLE patients as initial NP prodromes and/or in the preludes to their recurrent flares). In close collaboration with patient and clinician participants, we investigated NP symptom timings, symptom progression, potential (initial) disease and subsequent flare prodrome symptoms, and the consequences of under-identification. In addition, following D’Cruz’s clinical observation that nightmares often precede a flare in SLE patients,^15^ we investigated this theory quantitatively and qualitatively, including with other non-SLE SARD patients.

## Methods

### Participants and design

This study is part of the mixed methods INSPIRE research project. Data was collected in 2022 (with a shorter survey in 2023 to target under-represented groups) using the online survey instrument Qualtrics. Recruitment was via social media, online patient support groups and professional networks. For the INSPIRE project as a whole, patients had to be aged 18 years or over and report a diagnosis of any SARD confirmed on clinical correspondence. Diagnoses were not verified by the study team. For this component of the INSPIRE study, we only included survey data from SLE patients for the quantitative analysis. However, interview data was included from all SARD patients in order to explore the possibility of NP prodromes in SARDs as a whole. This included from patients with: inflammatory arthritis (IA), vasculitis, Sjögrens, systemic sclerosis, myositis, undifferentiated and mixed connective tissue diseases (UCTD and MCTD), and polymyalgia rheumatica (PMR).

SLE patients were asked to report when they had first experienced each of the listed NP symptoms (for those that they had experienced >3 times in their lives) in relation to the onset of their other SLE disease symptoms. Options included: over a year before, around the same time (within a year either side), and over one year after (further divided into 1–4 years and 5 years or more) onset of other (non-NP) SLE symptoms. Other quantitative data included timings of disrupted dreaming sleep in relation to hallucinations for those patients reporting experiencing these. Patients responding “I don’t know” were excluded from the analyses. Symptoms as opposed to diagnoses were used wherever possible to improve comparability between clinician and patient responses, and to reduce the influence of the previously identified difficulties in accurately diagnosing NP symptoms.^2^

Interviewees were purposively selected from INSPIRE survey respondents. This ensured that we acquired a range of views and experiences from participants with a broad range of disease and socio-demographic characteristics, including age and ethnicity. Interviews were conducted by three experienced medical researchers, usually via Zoom (or alternative remote technology), and were audio recorded and transcribed verbatim. Interviews were semi-structured and included questions on timings of NP symptoms, attributional views, and usual progression of symptoms in flares. In addition, nightmares were discussed and the term “daymare” was used to initiate conversations about possible hallucinations.

### Analysis

This component of the INSPIRE project was largely qualitative, with quantitative data being presented descriptively. The stages of thematic analysis^16^ of qualitative interview data included: 1) full immersion in the data; 2) developing a coding scheme, and subsequent coding; 3) combining participant transcript extracts for codes; and 4) members of the wider study team discussing and generating themes directly from the data. An additional analytical tool in line with our collaborative constructionist^17^ ethos of working with our participants was to discuss initial data, (anonymised) interview extracts, and initial theme ideas with subsequent interviewees. This enabled further breadth and depth of analysis, and reduced threats to validity by “participant/member checking”^18^ of analytical reasoning and initial themes. Further description of the methods is included in Supplementary information one.

### Ethical approval

Ethical approval was provided by The Cambridge University Psychology Research Committee: PRE 2022.027. Informed consent was taken electronically on surveys and verbally (audio recorded) for interviews. The pre-registered protocol and statistical analysis plan can be found at: https://osf.io/zrehm.

### Role of the funding source

The funder of the study had no role in the study design, data collection, analysis, interpretation, or writing of the manuscript.

## Results

A total of 1076 participants (n = 676 SLE patients and n = 400 clinicians) were surveyed, and 119 participants were interviewed (n = 69 SARD patients including 27 SLE patients, and n = 50 clinicians including 20 rheumatologists) (Table 1). Clinicians resided in a wide range of countries, with the largest group of survey respondents from the UK (45%). Patient survey respondents mostly resided in the UK (78%), followed by the rest of Europe (14%), and were predominantly female (94%).

**Table 1:** Participant characteristics.

Characteristic	Patient survey (n = 676) (%)	Patient interviews (n = 69) (%)	Clinician survey (n = 400) (%)	Clinician interviews (n = 50) (%)
Age (years)				
18–29	58 (9%)	6 (9%)	16 (4%)	0 (0%)
30–39	112 (17%)	6 (9%)	135 (34%)	11 (22%)
40–49	159 (24%)	17 (25%)	135 (34%)	19 (38%)
50–59	191 (28%)	16 (23%)	69 (17%)	12 (24%)
60–69 (60+ for clinicians)	109 (16%)	10 (14%)	45 (11%)	8 (16%)
70+	43 (6%)	14 (20%)	N/A	N/A
Prefer not to say	4 (<1%)	0 (0%)	0 (0%)	0 (0%)
Gender				
Female	634 (94%)	61 (88%)	209 (52%)	23 (46%)
Male	38 (6%)	8 (12%)	186 (47%)	27 (54%)
Other/undisclosed	4 (<1%)	0 (0%)	5 (1%)	0 (0%)
Country/region				
England	434 (64%)	39 (56%)	156 (39%)	28 (56%)
Scotland	48 (7%)	7 (10%)	16 (4%)	2 (4%)
Wales	32 (5%)	7 (10%)	6 (2%)	2 (4%)
N. Ireland or Republic of Ireland	15 (2%)	3 (4%)	2 (<1%)	0 (0%)
US or Canada	15 (2%)	4 (6%)	65 (16%)	4 (8%)
Europe	97 (14%)	4 (6%)	68 (17%)	6 (12%)
Asia	19 (3%)	2 (3%)	34 (9%)	3 (6%)
Latin America	2 (<1%)	0 (0%)	30 (8%)	4 (8%)
Australia or New Zealand	5 (<1%)	2 (3%)	10 (3%)	0 (0%)
Other	9 (2%)	1 (1%)	13 (3%)	1 (2%)
Ethnicity			Not recorded	Not recorded
White	542 (80%)	56 (81%)		
Asian	54 (8%)	7 (10%)		
Black	37 (5%)	4 (6%)		
Mixed	30 (4%)	2 (3%)		
Other	10 (1%)	0 (0%)		
Undisclosed	3 (<1%)	0 (0%)		
Disease			N/A	N/A
SLE	676 (100%)	27 (39%)		
Inflammatory arthritis		9 (13%)		
Vasculitis		3 (4%)		
Sjögrens		6 (9%)		
PMR		7 (10%)		
UCTD		9 (13%)		
Myositis		3 (4%)		
Systemic sclerosis		2 (3%)		
Mixed/multiple		3 (4%)		
Clinician role	N/A	N/A		
Rheumatologist			204 (51%)	20 (40%)
Psychiatrist			96 (24%)	8 (16%)
Neurologist			52 (13%)	10 (20%)
Rheumatology nurse			20 (5%)	4 (8%)
GP/Primary care physician			11 (3%)	5 (10%)
Other			27 (7%)	3 (6%)

Four themes and one subtheme were identified from the qualitative and quantitative data:

• Timing of NP symptoms in comparison to onset of disease.
• Progression of flare symptoms and pattern recognition, with the subtheme: NP symptoms as an early warning system.
• Disrupted dreaming sleep, nightmares and “daymares”: An example of a prodromal symptom.
• Impact of prodromal symptoms being unidentified/misattributed.

### Theme 1: timing of NP symptoms in comparison to onset of disease

Many rheumatologist participants stated that it was an “*established theory”* (multiple rheumatologists) that most attributable NP symptoms will present for the first time around the time of SLE diagnosis or disease onset, particularly psychosis (including hallucinations). Although we cannot make any attribution assertations from our data, several components of our findings contrasted with this prevailing view. This includes the timing of each NP symptom only first presenting at the same time (within a year) of other SLE disease symptom onset in approximately 1/5 to 1/3 of cases (Table 2). In addition, psychiatrist interviewees did not support the rheumatologists’ prevailing theory of timing of onset, and >50% of our SLE patient participants who reported having experienced hallucinations and/or delusions/paranoia first experienced them >1 year after other disease symptom onset.

**Table 2:** Timing of first occurrence of NP symptoms in relation to onset of non-NP SLE disease symptoms (as a % of those who have experienced the NP symptom >3 times in lifetime and completed this section of the survey) in SLE patients (n = 455 maximum).

Symptom (n)	BEFORE (>1 year) other disease symptom onset %	SAME time -within a year either side of symptom onset %	AFTER—Total. Calculated from (1–4 years + ≥5 years) %
Fatigue (n = 455)	47	38	15 (9 + 6)
Weakness/loss of strength (n = 293)	23	37	41 (19 + 22)
Feeling of unreality/disorientation (n = 221)	18	37	45 (20 + 25)
Cognitive dysfunction (n = 410)	18	36	46 (28 + 18)
Positive sensory symptoms (n = 297)	21	35	35 (21 + 24)
Tremors (n = 136)	15	33	52 (24 + 28)
Hallucinations (n = 106)	13	32	54 (27 + 27)
Hypersensitivity to noise/light (n = 314)	32	31	37 (22 + 15)
Severe headache (n = 280)	41	29	31 (13 + 18)
Negative sensory symptoms (n = 171)	24	29	47 (20 + 27)
Delusions and/or paranoia (n = 78)	18	29	52 (24 + 28)
Restlessness/agitation (n = 302)	27	29	43 (23 + 20)
Very low mood (n = 313)	34	28	38 (17 + 21)
Mania (n = 88)	27	28	44 (25 + 19)
Insomnia (n = 372)	28	28	44 (22 + 22)
Palpitations (n = 290)	20	27	53 (26 + 27)
Loss of coordination/balance (n = 273)	19	26	54 (27 + 27)
Difficulty swallowing (n = 180)	15	25	60 (22 + 38)
Uncontrollable emotions (n = 180)	30	25	45 (20 + 25)
Dizziness/raised heart rate on standing (n = 267)	31	25	43 (18 + 25)
Disrupted dreaming sleep (n = 272)	32	25	43 (23 + 20)
Disinhibition (n = 82)	33	24	42 (18 + 24)
Visual changes (n = 160)	21	23	56 (27 + 29)
Anxiety (n = 322)	39	22	40 (17 + 23)
Bowel or bladder problems (n = 302)	24	21	44 (23 + 31)
Tinnitus (n = 221)	23	21	55 (22 + 33)
Seizures (n = 36)	36	19	44 (8 + 36)
Obsessive thoughts or compulsive behaviours (n = 156)	36	17	46 (22 + 24)
Hearing loss (n = 101)	23	15	63 (24 + 39)

Note: Percentages may not add up to 100 due to rounding.

During interviews, most rheumatologists acknowledged that they did not have a medical explanation for this received wisdom, and the vast majority talked about timing of diagnosis as opposed to timing of symptom onset:

*“It is a mystery, we’ve no particular reasoning as to why we all say psychosis and hallucinations would occur near the diagnosis of lupus*. *I’m sure there’s a good reason because it’s well established but I don’t know the reason”* (Ppt 29, rheumatologist, England)

Psychiatrists stated that SLE patients experiencing a later onset of severe psychiatric symptoms than would be typical in a primary psychiatric disease (e.g., schizophrenia), where onset would generally be before mid-20s, was highly indicative of NPSLE. However, attribution can be complicated by the fact that some diseases may have a “psychiatric prodrome” as detailed in Table 3, and time of onset of SLE is often at the same age as usual onset of primary psychiatric disorders.

**Table 3:** Quotes contradicting the prevailing rheumatologist opinion that most attributable neuropsychiatric symptoms will present around diagnosis.

Timing of NP symptom onset	Illustrative quotes
Before diagnosis “psychiatric prodrome”	*…****psychiatric prodrome****, this is nicely illustrated in lupus, in MS, in Parkinson’s, in essence what you see is the first disruption, and the first presentation of the disease is the emotional symptoms, it’s the fatigue, you can’t sleep, you feel sad and tearful, you feel anxious and panicky. This is the direct effects of their disease … this depression is a marker of someone who is going to get MS 5 years later. Where lupus is much trickier is because age of onset of lupus tends to be much earlier, so someone in their 20’s, so that is around the same age someone may get their first psychotic episode, but in lupus that’s the disease causing it* (Ppt 115, psychiatrist, US)
Pre-disease onset of common NP symptoms	*You’re looking for the****temporal relationship****between the more definitive symptoms of lupus and the CNS symptoms … That would be strongly indicative, but I’d be a bit less reliant on saying the converse, so if they didn’t come on at the same time, does that mean you can definitively exclude them as a manifestation of lupus? No, you can’t, certainly something that is very common anyway like the depression* (Ppt 46, neurologist, England)
Symptom onset after diagnosis	*I don’t agree that we would be thinking that any psychiatric problems emerging later in life after the SLE diagnosis would be less likely to be SLE either as you do****lose biological resilience later in life****, so you’d be more prone to any of these neuropsychiatric problems as it’s the CNS that starts breaking down* (Ppt 73, psychiatrist, England)

Patients had often carefully considered their onset of NP symptoms in relation to the onset of autoimmune disease to assess attribution. Some patients self-attributed NP symptoms directly to their SLE or other SARDs due to their absence prior to disease onset:

*“I get hypomania which I believe is directly due to SLE … I have previously had diagnosis such as "affective disorder" bipolar type II and it took several years, and several different psychiatric professionals involved in my care before anyone professional thought my mental health issues are very likely due to neuropsychiatric SLE … I have never had neurology or brain scan investigations, but I always had stable "normal" mental health prior to having SLE symptoms”* (Ppt 1395, SLE, England)

Conversely, other patients cited long-standing pre-existing psychiatric symptoms as being evidence against direct and/or indirect attributability to their SARD.

*“The major depression is definitely not from coping with an autoimmune illness. I did not even have the autoimmune illness in [year] … when I had my first episode of major depression. I think they are two distinct illnesses”* (Ppt 1306, multiple SARDs, US).

### Theme 2: progression of flare symptoms and pattern recognition

Some SLE patients could detail their usual progression of symptoms in a flare, and several of those with long-standing disease highlighted the point at which they would require increased immunosuppression and/or other treatment. Two examples of patients’ descriptions of their usual progression of symptoms in a flare are detailed in Table 4. Of note, the participant with lupus nephritis, in common with many other patients not reporting a diagnosis of NPSLE, also experienced several neuropsychiatric symptoms at various stages in their flares. Similarly, participants with NPSLE usually had several non-NP symptoms intermingled in their progression of symptoms.

**Table 4:** Examples of two SLE study participants’ progression of symptoms in a flare.

	Participant 164, SLE, England	Participant 1312, SLE, Wales
Diagnosis	Lupus nephritis (biopsy-proven)	NPSLE (clinical assessment)
Usual progression of symptoms in a flare	1. Increased fatigue 2. Sudden onset of low mood (Ppt described how this differs in intensity and feelings from the more “reactive” low mood from feeling unwell that sometimes came at a later stage of a flare) 3. Joint pain 4. Protein in urine 5. Nightmares 6. Malar rash 7. Hallucinations (mild, not unpleasant, visual “*usually small animals*”). Ppt had never reported this to clinicians	1. Feeling “*over-excited”* and “*unable to settle*” (Interview transcript assessed by study psychiatrists as likely mania) 2. Insomnia 3. Increasingly talkative 4. Labile mood and uncontrollable emotions (description assessed by study psychiatrists as likely pseudobulbar affect) 5. Cognitive dysfunction (worsening as flare progresses) 6. Joint pain 7. Severe fatigue 8. Difficulty swallowing and nausea 9. Joint swelling including visibly in the knees and hands
Advanced stages of a flare when untreated	Had previously progressed to patient’s usual later flare symptoms which include pericarditis and nephritis.	Participant reported that symptoms in several flares had progressed to severe confusion and delusions when not treated at an earlier stage “*There were people on the TV trying to kill me*”.
Laboratory results (patient-reported)	Flare usually confirmed in laboratory tests including raised anti dsDNA and reduced eGFR.	Ppt was unsure of laboratory test results and had never had brain imaging.

During interviews, patients often differentiated among different types, timings, and potential causes of the same symptoms. For example, several patients described how they felt that some types of depressive symptoms were directly attributable to active inflammation due to its time of onset, and differences in type and intensity compared to their more “reactive” low mood that could be more attributable to a consequence of psychological distress. Many patients also described a type of sudden severe “*hit like a truck*” (Ppt 52, UCTD, Canada) fatigue coinciding with flare activity which they could differentiate from physical deconditioning or the more chronic fatigue that many experienced indefinitely. Very few felt that they had been given the opportunity to voice these opinions to their clinicians.

Other patients had not identified their usual progression of SARD symptoms, or considered the potential connection among multiple NP symptoms, until it was explored in interview. Most clinicians (aside from most psychiatrists and nurses) reported limited available clinic time for eliciting and discussing symptom progression. As the physician in the quote below details, symptom progression in NPSLE flares had high levels of inter-patient variability. However, symptoms often progressed in a similar manner in future flares in each individual, thus potentially aiding quicker treatment in subsequent NPSLE flares if monitored:

*“As far as neurolupus generally, I think what you do find is that it’s important to talk to patients about what leads up to a flare in them, so they and we know. In my own experience it’s quite idiosyncratic, different people have different things … they know much more about themselves than I do”* (Ppt 13, vasculitis specialist)

### Subtheme: NP symptoms as an early warning system

Multiple diverse NP symptoms were recognised by some patients from all SARD groups as the earliest indicators of an impending flare:

*“The first signs will be mental or neurological and if I choose to ignore these, I then delay the period that follows for recovery”* (Ppt 83, Sjögrens, Wales)

Patient self-monitoring of increases in symptom range and frequency that may indicate an impending flare often incorporated symptoms that were absent from current diagnostic guidelines and only rarely identified by clinician interviewees as related to SLE/NPSLE, yet were found to be common when explored in patient interviews. These NP prodromal symptoms were reported as sometimes preceding the more widely recognised SLE and other SARD symptoms such as joint pain, rashes, and other organ involvement.

A diverse range of symptoms were discussed including: sudden changes in mood (usually a lowering, but several patient interviews were assessed by our study psychiatrists as likely describing elevated mood/mania), increased nightmares, a “feeling of unreality”, and/or increased sensory symptoms. For example, one SLE patient reported increased positive sensory symptoms in the week preceding all of her psychotic episodes. These types of widespread sensory symptoms were commonly reported as preceding flares, yet non-localisable weakness or sensory symptoms were considered by some neurologist participants to sometimes be more as a result of hypervigilance (“over-attention”) than an actual increase in symptoms. Other clinicians hypothesised that increasing neuro-inflammation as a flare progresses would often cause these multiple diffuse symptoms.

### Theme 3: disrupted dreaming sleep, nightmares and “daymares”: an example of a prodromal symptom

Although many patients only considered the potential link between increasing nightmares and flares during the interview following direct questioning, other patients had already recognised nightmares often preceded their flares: “*usually when I’m either in a flare or just before one”* (Ppt 1730, SLE, England), and others had attributed increasing nightmares to being unwell:

*“Horrific, like murders, like skin coming off people, horrific … I think it’s like when I’m overwhelmed which could be the lupus being bad … So I think the more stress my body is under then the more vivid and bad the dreaming would be”* (Ppt 1159, SLE, Ireland)

Several SLE patients recounted flares consistently involving the segueing of increasingly vivid and distressing nightmares into distorted reality and daytime hallucinations. These qualitative findings were also demonstrated quantitatively with 61% of SLE patients (compared to 36% of all other SARDs combined) experiencing hallucinations reporting increasingly disrupted dreaming preceding their hallucinations, 2% reporting less disrupted sleep, and 36% reporting no change.

Types of nightmares varied (examples can be found in Table 5), and descriptions of flare related nightmares often involved being attacked, trapped, crushed, or falling. The interviewers used the discussions of nightmares and the term “daymare” to gently introduce discussing hallucinations. This not only reduced participant reluctance to share these experiences, but also was reported to be a “lightbulb” (i.e., episode of enlightened insight) moment. The description of feeling: “*in-between asleep and awake*” (Ppt 9, SLE, Scotland) was not infrequent, including for hallucinations occurring in the daytime when fully awake. Several participants had not recognised these episodes as hallucinations, and often used sleep terminology, such as “*waking dreams*” (Ppt 704, SLE, England) to describe them:

*“Like I’m asleep and dreaming but then I’m not actually asleep but what I see is kind of like a dream where things are changing quickly”* (Ppt 1372, multiple SARDs, England)

**Table 5:** Selection of INSPIRE study participant quotes describing nightmares and “daymares”.

Type (in descending order of frequency of type reported)	Selected patient quotes
Frequent descriptions of being crushed or trapped	*A lot about falling and not landing, ones where I can’t breathe and where someone is sitting on my chest, being somewhere scary and not being able to get out* (Ppt 69, SLE, England)
Recurring frightening dreams where the patient or their families are the victim(s)	*They are usually quite frightening, like there’s a serial killer after me and the last few years I have the same one … if [it] was like the serial killer one and he’s got my legs or something I can still feel something on my legs even when I’m then awake* (Ppt 52, UCTD, Canada)
“Daymares”	*[When] you said that word daymare and as soon as you said that it just made sense, it’s like not necessarily scary, it’s just like you’ve had a dream and yet you’re sitting awake in the garden … I see different things, it’s like I come out of it and it’s like when you wake up and you can’t remember your dream and you’re there but you’re not there … it’s like feeling really disorientated, the nearest thing I can think of is that I feel like I’m Alice in Wonderland* (Ppt 132, SLE, England)
Violent dreams where the patient is the perpetrator	*I have lots of violent dreams … one of them was somebody attacking me and I ended up slitting their throat. Oh I mean, really nasty. I mean I'm not a violent person at all. I don't even kill an insect … I’d be riding a horse, going around cutting people out with my sword, that kind of thing, which is bizarre. And I came to the conclusion that that's probably me fighting my own system, is probably all autoimmune, it’s somehow subconsciously speaking through. I'm probably attacking myself, that's the only thing I can logically make sense out of it.* (Ppt 978, SLE, England)

Although many of the other specialities reported discussing sleep disruption with patients, only one of the rheumatologist interviewees had previously considered nightmares as potentially related to SLE flares. Several rheumatologists voiced scepticism about this hypothesised relationship, although the majority stated that they would ask SARD patients about nightmares and “daymares” more often in the future after being shown evidence of an association between nightmares and hallucinations. There was agreement that recognising and eliciting these early flare symptoms may improve care and even reduce clinic times by averting flares at any earlier stage, although some rheumatologists were clear that limited appointment times meant that these symptoms would not be prioritised for discussion:

*“I hear what you are saying and what David D’Cruz is saying about the nightmares and hallucinations, and I believe it, but what I am saying is that you cannot conceivably include that as well as the routine management of lupus”* (Ppt 4, Rheumatologist, England)

### Theme 4: impact of prodromal symptoms being unidentified/misattributed

Many patients reported having been (mis)diagnosed with a separate psychiatric and/or psychosomatic condition prior to their SARD diagnosis, and several surmised that the similar timings and improvement following SARD treatment seemed unlikely to be coincidental:

*“At 18 I was diagnosed with borderline personality disorder, and then 6 months later with lupus at 19, so it’s all very close together and it was strange that when my BPD got under control and my lupus got under control was within 6 months”* (Ppt 9, SLE, Scotland)

Several patients had been initially hospitalised in their late teens/early 20s with psychotic illness and/or suicidal ideation, discovered retrospectively to have been their presenting SARD (particularly SLE) symptom. Clinician participants also detailed several cases where patients with undiagnosed SLE had been involuntary psychiatric inpatients for many months before any autoimmune panels were requested:

*“I’ve seen them admitted for an episode of psychosis and the lupus isn’t screened for until someone says “oh I wonder if it might be lupus” … but it was several months and very difficult … especially with young women and it’s learning more that that is how lupus affects some people and it’s not anti-psychotic drugs they needed, it’s like a lot of steroids”* (Ppt 6, Nurse, Scotland)

Early misattributions of SARD symptoms to primary psychiatric or psychosomatic conditions were frequently reported to have delayed SARD diagnosis and led to future misattributions.

## Discussion

We investigated the timing and progression of neuropsychiatric symptoms in SLE initial disease onset and recurrent flares, and carried out exploratory qualitative analyses of NP symptom timings in other SARDs. Of importance was the finding of a possible NP prodrome in recurrent flares in some patients which could provide more reliable and timely predictions of impeding flares, and could thus help indicate when to initiate/increase immunosuppression. Most SLE patients experienced varied combinations of NP and non-NP symptoms in the early stages of the disease and recurrent flares. This highlights the importance of ascertaining and monitoring each patient’s usual progression of all symptoms, as early non-NP (e.g., malar rash) symptoms may predict an NPSLE flare in some patients. Conversely, NP symptoms may warn of an impending flare involving other organs, e.g., lupus nephritis.

In initiating this study, we proposed two main criteria that would ensure the identification of an NP prodrome in SLE would be most useful in clinical practice: 1) within-person similarity of prodrome symptoms in recurrent flares, and 2) a degree of commonality of prodromal symptoms among patients. Our data provided qualitative exploratory evidence for criterion 1, and also demonstrated that some symptoms such as cognitive dysfunction, nightmares, and sensory symptoms were commonly experienced by many SLE (and other SARDs) patients as an NP prodrome (criteria 2). However, there was considerable inter-person variation in prodromal and progression of symptoms. A prospective quantitative study is therefore now required to test the theory of an NP prodrome in SLE and further explore NP symptoms in other SARDs.

This study was initiated due to concerns regarding the high frequency of misattributions of early SLE and other SARD NP symptoms leading to suboptimal or incorrect treatment, persisting psychological damage and distrust in clinicians.^7^ A potential cause of misattribution is the assessment of NP symptoms as less/non-attributable to SLE if they did not first present within a limited timeframe around the time of initial confirmed diagnosis.^4^^,^^6^^,^^19^ Our results suggested that many patients experienced their first episode of many NP symptoms >1 year after onset of their other SLE symptoms. This includes hallucinations or delusions/paranoia, which conflicts with the prevailing theory cited by many of our rheumatologist participants and in some research,^20^^,^^21^ that these symptoms usually occur around time of diagnosis or disease onset. Although immunosuppression following diagnosis for other SLE disease manifestations may confer a protective effect (e.g., hydroxychloroquine reducing seizure incidence^22^), and reduce the chances of developing later onset NP symptoms, clinicians and patients should remain vigilant for NP symptoms first presenting at *any stage* in the disease course. Excluding symptoms occurring at various timescales before diagnosis as unrelated to SLE^6^^,^^19^ will also exclude symptoms that may represent a psychiatric and/or neurological prodrome^23^ to SLE.

The differences in frequencies of each symptom’s first occurrence has multiple possible explanations, including that symptoms common in the general population, such as anxiety and headache, will be more likely to have been experienced prior to disease ascertainment. A possible explanation for the relatively high proportion of first episode seizure preceding the onset of (other) SLE symptoms may support the hypothesis that systemic immune activation, driven by the production of proinflammatory cytokines, can lower the seizure threshold and trigger seizures prior to the manifestation of other disease symptoms.^24^

A neuropsychiatric prodrome has been reported in multiple diseases, including MS,^11^ several dementia syndromes,^9^ and PD,^10^ with symptoms potentially attributable to early (often undetectable using conventional neuroimaging) CNS involvement antedating the more familiar full syndrome stages. Prodromal symptoms reported in the literature include anxiety and mood symptoms, cognitive dysfunction, disinhibition, psychosis, personality changes, and sleep disturbances.^9^, ^10^, ^11^ Many of these documented prodromal symptoms in other diseases were also experienced by our SLE and other SARDs participants, yet they were rarely reported to clinicians. This under-reporting may partially explain why the prodromal literature is limited in SLE, although there are case reports of initial SLE symptoms being psychiatric, including mania.^25^ Euphoric/manic episodes preceding both first episode NPSLE and antedating recurrent flares were also reported by some of our participants. As these episodes are less easily conceived of as a “natural reaction” to chronic unpredictable illness and are typically more dramatic/disruptive in presentation than depressive states, manic episodes can be easier to attribute directly to the SLE than the more common depressive episodes.

Although depressive and anxiety symptoms (which can be subsyndromal for full spectrum disorders) are also commonly experienced as a reaction to increased disease activity (such as in a manifest SLE flare), experiencing these symptoms *preceding* a flare is potentially more suggestive of the direct impact of an incipient SLE flare on the brain, not a “reaction” to the flare. This reasoning may apply likewise with sensory symptoms, whereby prodromal/early flare sensory symptoms are less logically attributable to the theory that some patients pay additional attention (“hypervigilance”)^26^ to normal bodily sensations when experiencing increased pain and other disease symptoms, as the sensory symptoms may antedate a manifest SLE flare. Moreover, as these symptoms appear to be early indictors of impending flares for some patients, an appropriate level of vigilance and monitoring of progression of symptoms can assist in identification of flares at an early stage.

We used quantitative and qualitative methods to test D’Cruz’s clinical observation that nightmares often preceded SLE flares,^15^ and identified that the majority of SLE patients (excluding those who could not recall their progression of these symptoms) who experienced hallucinations had increasingly disrupted dreaming sleep preceding their hallucinations. Nightmares are characterised by intense dysphoric dreaming that arise primarily from rapid eye movement (REM) sleep. Whilst a normal phenomenon, they are seen in increased frequency in a range of neurological and psychiatric disorders, such as Parkinson’s disease, narcolepsy, acute stress disorder, posttraumatic stress disorder, and psychotic disorders. The underlying pathophysiology of nightmare disorder remains unelucidated, and there remains the question as to whether nightmares represent a mechanism for an adapted response to threat in wakefulness or are symptoms of underlying clinical conditions.^27^ Certainly, nightmare disorder seems more common in conditions that influence REM stability and in psychiatric disorders associated with a heightened response to threat. Thus, putative mechanisms explaining this possible association between NPSLE and nightmares could include inherent effects on REM sleep stability and sleep architecture specific to the SLE or, less specifically, to generalised inflammation, or attributable to neurophysiological changes as a result of SLE CNS involvement.

Initiating discussion of NP symptoms by first discussing disrupted dreaming sleep, and using more descriptive and less stigmatised language such as “daymares” rather than “hallucinations,” may encourage more patient openness in reporting these often feared and stigmatised symptoms. Indeed, several rheumatologist participants gave feedback to the research team some months after their interviews of vastly increased proportions of SLE patients reporting hallucinations and nightmares due to direct yet non-judgemental use of the suggested “nightmare/daymare” terminology.

Although symptoms such as increasing nightmares,^15^ sensory disturbances, or sudden changes in mood were often not prioritised by clinicians and patients for discussion and monitoring, this study demonstrates their potential importance in their being a frequent precursor to more “tangible” disease activity. Identification of SLE/SARD patients at “risk of imminent flare”^28^ could therefore be improved by valuing every patient’s “experiential knowledge”,^29^ “pattern recognition”,^2^ and “attributional insights”,^2^ in combination with monitoring “preflare” biomarkers,^28^ and greater use of AI.^30^

It is also important to consider the increasing evidence that an episode of severe psychiatric illness, an infection, or a stressful life event, may *induce* immune dysregulation and thus precipitate the onset/flare of an autoimmune disease.^31^ This complicates any simple model of attribution, in so far as rheumatic disease activity and psychiatric illness may *both* be caused by potent environmental stressors, rather than existing in any more simplistic bidirectional relationship to each other. This makes it challenging to distinguish between whether a NP symptom is a first manifestation of the disease/flare or a NP symptom that is a consequence of another condition that in turn has activated autoimmunity. In addition, patients with severe SARDs are often treated acutely with high dose corticosteroids or other immunomodulators, thus immunomodulator-induced psychiatric disorders must also be considered. Moreover, multiple additional confounding factors, such as pre-existing psychiatric illness, medication use (although the survey specified that patients should not include NP symptoms that were considered to be a direct effect of medication use), and psychosocial contributors, should be accounted for in any subsequent quantitative study, and alternative causes of psychiatric symptoms fully explored.

General strengths and limitations of the INSPIRE project are detailed in Supplementary information 1. Of particular relevance is the adverse influence of recall bias,^32^ particularly in patients remembering the first episode of each symptom in relation to disease onset, and anchoring bias where they may have been overly influenced by their initial perception of the diagnosis/misdiagnosis at the time. In addition, the self-selecting nature of online surveys will result in a study population that is not wholly generalisable, particularly in relation to under-served communities. Diagnoses were also unable to be validated, although we asked for the diagnosis/diagnoses as written on their clinic letter and excluded participants reporting uncertain diagnoses. We also excluded patients under the age of 18, and this age group may have a higher NP disease burden and earlier onset of NP symptoms in their disease course. For example, Appenzeller et al. showed that the majority of juvenile SLE patients have neuropsychiatric events in the first 2 years of disease with 30–70% of patients presenting with multiple neuropsychiatric events through disease’s course.^33^ Moreover, we have been unable to differentiate between whether some NP symptoms occur in a pre-disease prodromal stage or whether they are simply early symptoms of the disease/flare. In addition, we only asked patients for the timing of their symptoms for those symptoms experienced >3 times in their lives, reducing the direct comparability with previous research.

Participants who marked “I don’t know” in the nightmare/hallucination association may be more likely to be in the “no change” category as the “more/less” categories would be more memorable. Thus, the observed association is likely to be inflated due to these participants being excluded. However, this bias would apply to all SARDs so the comparative percentages of increasingly disrupted dreaming sleep preceding hallucinations (61% SLE vs 36% other SARDs) may indicate a more SLE specific prodrome which requires testing prospectively. It is possible that some patients may have described hallucinations in the context of focal seizure activity,^34^ although both are part of the spectrum of NPSLE. It is also impossible from our patient-reported data to ascertain if the higher than anticipated prevalence of symptoms such as visual or hearing loss was directly related to their SARD or other mechanisms of effect including age-related sensory impairment. Study strengths include our study team’s development of mutually trusting relationships with patient participants leading to rapport in interviews and openness with sharing their often stigmatised and distressing symptoms, many of which had not been reported to clinicians, and thus remain widely under-recognised. An additional strength is the team’s willingness to challenge the perpetuation of “established theories” in both research and in practice when they may be adversely impacting patient care and the medical rationale is unclear.

In conclusion, neuropsychiatric symptoms may first present at *any* stage in the disease course of SLE, including in the period before a formal validated diagnosis of SLE. Our findings of an NP prodrome providing an “early warning system” of disease onset and impending flares in some patients may enable earlier and more accurate identification and treatment of NPSLE/SLE (and potentially other SARD) flares. A key point is that patients’ brains in the early stages of disease may express psychiatric as well as neurological symptoms, often before physically manifest symptoms are clinically apparent. Prodromal symptoms differed among individuals but often followed a similar pattern of progression in an individual’s recurrent flares. This exploratory qualitative finding requires further testing using quantitative methodology. Increased clinician-patient collaboration in ascertaining, documenting and monitoring each SARD patient’s usual progression of symptoms in a flare could enable more timely intervention.

## Contributors

MS and DD'C conceived of the idea for the INSPIRE project and acquired funding. MS, RH, MU and JB collected the data. MS, RH and FN advised on methodology and study design. MS, RH, MP, GL, TP, LA and AB reviewed the relevant literature. MS, RH, MB, CB and FN analysed the data, with input and discussions with all other co-authors. MS and JB have directly accessed and verify the underlying data reported in this manuscript. DD'C supervised the study. MS wrote the original draft with substantial contributions from JAB, GL, TAP, MP, RH, MB, and AB. All authors attended meetings and discussion groups including on analysing, discussing, and agreeing qualitative themes. All co-authors reviewed and edited the final manuscript and earlier drafts.

## Data sharing statement

Anonymised data will be available on reasonable request following the completion of the INSPIRE studies.

## Declaration of interests

MS was funded by The Lupus Trust for this study via funding provided to Cambridge University. JAB has received speaking fees from Psychiatric Times and Oakstone and receives royalties from American Psychiatric Publishing, Springer International, Lippincott Williams & Wilkins, and Cambridge University Press. LA has received consultancy fees/speaker fees from: Eli Lilly, Glaxo Smith Kline, Janssen, Novartis, Pfizer, UCB, and Werfen Group, CG reports consultancy/advisory fees from: Alumis, Amgen, Astra-Zeneca, Sanofi, UCB and MGP. DD’C reports consultancy/speaker fees from GSK, Eli Lilly, Vifor and UCB, and a leadership role on the board of APS support UK. All other authors declare no potential conflicts of interest. TP was supported by the National Institute for Health Research (NIHR) Biomedical Research Centre at South London and Maudsley NHS Foundation Trust and King’s College London. This paper represents independent research part-funded by the National Institute for Health Research (NIHR) Biomedical Research Centre at South London and Maudsley NHS Foundation Trust and King’s College London. The views expressed are those of the study participants and/or author(s) and not necessarily those of the NHS, the funders, the NIHR or the Department of Health and Social Care.
