# Supplementary material for: Neuropsychiatric prodromes and symptom timings in relation to disease onset and/or flares in SLE: results from the mixed methods international INSPIRE study
Source: eClinicalMedicine. 2024 May 20;73:102634. doi: 10.1016/j.eclinm.2024.102634 (PMC11490656; doi:10.1016/j.eclinm.2024.102634)
Supplement: Supplementary information [file mmc1.docx]

**Supplementary Data S1- Additional methodological information for the INSPIRE research studies**

**Contents**

| **Section** | **Page number** |
| --- | --- |
| 1. Study design including Figure S1- A1 – study design flowchart | 1 |
| 1. Survey design | 3 |
| 1. Symptom list and terminology | 3 |
| 1. Recruitment | 5 |
| 1. Data cleaning | 5 |
| 1. Interviews | 6 |
| 1. Analysis | 6 |
| 1. Research team and reflexivity | 8 |
| 1. Additional limitations | 9 |
| 1. COREQ checklist | 10 |
| 1. Supplementary additional references | 11 |

1. **Study design**

The INSPIRE (Investigating Neuropsychiatric Symptom Prevalence and Impact in Rheumatology patient Experiences) research project encompasses a series of inter-related studies exploring various aspects of SARD NP symptoms, including: prevalence and identification, impact on patient lives, eliciting and reporting of NP symptoms, and attribution. Additional studies will be looking in detail at individual symptoms including hallucinations, cognitive dysfunction and tinnitus, and at individual sub-group experiences and potential SARD inequalities related to characteristics such as race and gender.

The INSPIRE research project uses mixed methods to allow for in-depth exploration and further explanation of the quantitative data received from the surveys, and for the pre-survey interviews to inform the direction of the surveys. Integration throughout attempts to reduce the weaknesses that can arise from research collecting and analysing either qualitative or quantitative data^1^ ^,^ and highlights the human dimension behind the statistics. The study design flowchart is at Figure A1, and demonstrates the level of data integration at each stage in the research process. Prior to the main separate and integrated analyses, interim analysis are carried out to inform the content of subsequent interviews. Aside from the first interviews, the majority of interviewees were provided with study findings in the form of graphs, figures and/or anonymised quotes from earlier interviews. This enabled us to gauge clinician’s and patient’s reactions to the findings and the views of the other party in medical relationships, and for these to be included within the qualitative analysis.

We use combined moderate realist ^2^ and constructivist ^3^ paradigms for qualitative research as this allows for multiple interpretations of a shared reality which we can gain better knowledge of, and can then use this knowledge to change and improve that reality and individual experiences of it. This also fits in best with our ethos of working with participants to construct the themes as they arise. We are as inclusive as possible and aim to represent the collective voice of participants, whilst also analysing and incorporating divergent views. We ‘member check’ ^4^ by sending draft results and later manuscripts to multiple study participants for agreement, suggested changes and improvements. We carefully consider all suggestions whilst making efforts to ensure our final analyses/report remains true to the data acquired. Key aims of all our studies are to represent the views/experiences of these patient and clinician groups, to give them a wider voice, improve understanding and medical relationships, and further their interests, whilst ensuring they are protected from harm. This includes ensuring confidentiality was maintained, ethical standards were clarified and maintained, and all interviews followed the research care ethic ^5^.

Note: This study team carries out multiple rheumatology research studies using the same mixed methods paradigm and procedures. Therefore, relevant sections of the supplementary methods section are copied with permission from previous supplementary information published by Rheumatology and Rheumatology Advances in Practice.

**Fig S1-A1 – Study design flowchart demonstrating the integration of methods at each stage.**

**White represents purely qualitative methods, black represents purely quantitative methods, with varying shades of grey representing differing dominance of either method at each stage)**

**Quantitative data analysis**

Descriptive and inferential

**Qualitative**  **data analysis**

Thematic

**Integrated** **data analysis**

Included exploring convergence or divergence of results, using qualitative data to explain/ add depth to quantitative results and triangulation

Additional **surveys** to increase representation and further explore early findings

Additional **interviews** to further explore preliminary findings

**Presentation** of results

1. **Survey design**

The surveys were designed with input from multiple patients, clinicians and rheumatology charities so as to include questions thought to be most relevant. Exploratory questions were included in our previous research studies ^6^ to assist in informing the development of the INSPIRE study direction and content. Pre-INSPIRE survey interviews were used to further explore issues concerning SARD NP symptoms including: identification, attribution and management, and the broader topics of medical relationships, trust and wider views of stigma. Additional clinicians, patients and patient support group leaders were then consulted to further refine the potential symptoms to include and how to phrase. Pilot surveys were then completed by clinicians (n=111) and patients (n=416) to incorporate a broad range of NP symptoms identified by patients as important as opposed to just those reported in the current literature. Discussions were then initiated on social media patient groups to ascertain views and patient descriptions of NP symptoms and the symptom list was added to.

An INSPIRE study neurologist (MZ) and psychiatrist (TP) advised on the medically correct phrasing of symptoms, and patients were then consulted to ascertain approval and understanding. Patient suggestions were used for phrasings of those symptoms which were discussed as common yet difficult to articulate. These were checked for medical accuracy by the study clinicians, and the clinical, lay terms and a description where necessary included in the surveys (e.g. disorientation, feeling ‘spaced out’). Wherever possible, symptom descriptions were used, as opposed to diagnoses. For example, very low mood rather than depression to aid comparability between patient and clinician reports, and to reduce the inaccuracies from relying on official diagnoses and/or self-diagnoses. Surveys explicitly requested that attribution considerations not be included when making prevalence estimates.

Most response options were on a 5 or 7 point Likert type scale, with 0 being the lowest (e.g. Having not experienced that symptom at all or the lowest opinion of care) to 4 (or 6) being the highest (e.g. experiencing that symptom all the time or the highest opinion of care). Quality of life, adapting to disease and satisfaction with care were generally measured on a scale of 0-100. Clinicians’ estimates of prevalence and attribution were on a slider of 0-100 to signify percentages.

The survey was trialled initially by using the ‘think aloud’ cognitive interviewing technique ^7^ (with n=3 clinicians, n=5 patients and n=1 control) where survey respondents complete the draft survey whilst talking through with the interviewer (via Zoom or telephone) their understanding of the questions and reasoning when answering. This is to try and ensure questions are interpretated as intended and ascertain any areas of potential misunderstanding or confusion. Several questions were then rephrased to reduce the misunderstandings identified. For example, 2/3 of the clinician ‘think aloud’ respondents expressed concern about other clinicians misunderstanding the prevalence questions to include attribution considerations. Therefore, in the final survey design, this question was simplified and the instruction to not include attribution views was written in bold. However, several clinicians in later interviews acknowledged that their low prevalence estimates may have been due to subconsciously incorporating attribution estimates into their interpretation.

Due to the high number of symptoms being elicited on the patient survey, these were randomly ordered by Qualtrics for each participant to reduce bias in answering earlier questions more accurately and becoming fatigued or bored at the end of a long list. There were multiple follow-on questions for each symptom, including: impact on life, views on attribution, timing in relation to other symptom etc. These will be used in the various INSPIRE study papers. Due to the length of the survey (30 minutes to > 3 hours), patient participants were given the opportunity to stop and return at any stage, or have the rest of the survey emailed later to ensure fatigue did not impact later responses. The clinician survey was much shorter (approx. 15-25 minutes).

The survey was made available internationally in English. Several participants responded to free text questions in their language. This was then translated into English for transferral to Nvivo and analysis.

1. **Symptom list and descriptions as presented to patients, controls and clinicians.**

Very low mood (from more so than normal feelings of being sad or ‘down’ to feeling severely depressed).

Anxiety (more so than everyday worries).

Mania/ euphoria (feeling overly happy and/or overly excited or ‘hyper’).

Obsessive thoughts and/or compulsive behaviour (intrusive thoughts which are difficult/beyond control and/or repetitive behaviours as a result).

Uncontrollable and/or inappropriate emotions or emotional behaviours (e.g. laughter, crying, anger for no external usual reason).

Hallucinations (see, hear, smell or feel things that aren’t real or things appear different to the reality)

Delusions – strongly believe in something that is untrue despite having evidence to the contrary and/or Paranoia – unduly suspicious or paranoid of others.

Loss of inhibition or ‘filter’ in doing or saying things that may not be socially acceptable.

Suicidal thoughts.

Weakness/ Changes in strength in the limbs (not from fatigue or pain).

Negative sensory symptoms - lack of sensation in arms and/or legs (not just from temporary body position).

Positive sensory symptoms – e.g. tingling, pins and needles feeling in head, arms and/or legs (not just from temporarily trapping that nerve by body position).

Bowel or bladder symptoms (urgency, incontinence, increased frequency or inability/ difficulty in passing water).

Visual changes. Visual loss, visual changes and/or visual disturbances (e.g. visual snow, light flashes, after images). Do NOT tick please if you had a migraine at the time and/or all symptoms are correctable with glasses or are from dryness or floaters).

Loss of coordination and/or balance.

Tinnitus (the perception of a ringing or other sound in the ear or head that doesn’t come from an external source).

Dizziness/fainting on standing, raised heart rate and/or decreased blood pressure on standing.

Hypersensitivity to noises/light etc.

Fatigue (much more than normal tiredness, out of proportion to level of activity, doesn’t fully improve with rest).

Severe headaches (where normal painkillers don’t resolve it), including migraines.

Cognitive problems (difficulties thinking, talking, planning, remembering – sometimes known as ‘brain fog’) More severe than the everyday cognitive/memory slips everyone gets.

Insomnia (difficulty getting to sleep or staying asleep).

Disrupted dreaming sleep (Nightmares, vivid dreams, lucid dreaming (where you know you’re in a dream and can have some control), Sleep paralysis and/or acting out your dreams whilst you’re asleep.

Seizures/epilepsy.

Hearing loss (not age-related decrease).

Tremors.

Feelings of unreality, feeling disorientated or ‘spaced out’.

Restlessness/agitation/’can't settle’.

Difficulty swallowing.

Palpitations and/or fast heart rate.

**Selection of terminology and symptoms for inclusion**

Due to most symptoms having multiple potential aetiologies, study inclusion was on pragmatic and phenomenological grounds (e.g. classifying as neuropsychiatric to distinguish from dermatological, musculoskeletal, or respiratory symptom groupings), and following pre-survey consultation with patients and clinicians rather than to represent any fixed notion of aetiology or mechanism. We explored self-reported mood states in patients, both elevated/euphoric mood and depressed/sad mood states, which may or may not reflect full spectrum bipolar or depressive disorders *per se* on formal diagnostic interview.

There is also no consensus in the literature of an accepted term that encompasses all the neuropsychiatric experiences of these patients. After extensive consultation with patients and clinicians, the term “symptom” was selected as this was the most familiar and widely understood. We will continue to use this term throughout the INSPIRE studies in order to ensure consistency and conceptual coherence between the survey and interview terminology and the analyses. This has a limitation in that some “symptoms” may be classified in other research and care as “syndromes”, “manifestations” or “events”, and terminology may have different connotations between and within specialities, and between patients and clinicians.

Although the inclusion of fatigue was debated due its aetiology being likely highly heterogenous, and it being excluded from previous NPSLE guidelines and algorithms ^8-10^, justifications for inclusion were: 1) It is increasingly pragmatically classified as a neuropsychiatric symptom in other research into complex multi-system conditions, such as long-Covid ^11^. 2) It is the most impactful systemic autoimmune rheumatic disease (SARD) manifestation ^12^, and therefore any additional evidence as to level of direct/indirect attributability could be of great value in determining the best treatment, and 3) Consultations with patients during study design revealed a strong preference for its inclusion.

The prevalence question for clinicians was phrased as below:

PROPORTION of lupus patients with each manifestation.

Please move the sliders to what you feel is the percentage of lupus patients (OVERALL, not just your own) who will **ever** have experienced (in their lives) each mental health/ Neuropsychiatric symptom listed below **(regardless of whether or not it can be attributed to the disease).** Descriptions are those used on the patient survey to aid statistical comparability. For all these symptom questions, please do not include those patients with that symptom occurring ONLY when under the influence of recreational drugs, alcohol, during an infection or as a medication side-effect.

1. **Recruitment**

Patient recruitment to the study was by open invitation internationally by provision of an information sheet and online link to a survey using Qualtrics (an online questionnaire platform) on multiple disease online forums, predominantly via HealthUnlocked, Facebook groups and international charities. The patient survey was available for two months from Jul-Sep 2022. Clinician participants were primarily recruited via rheumatology, neurology, psychiatry and primary care networks, including using twitter and International and regional professional groups. The study was supported by multiple SARDs charities, and the British Society for Rheumatology and advertised through their networks and by Rheumatology journal. The Association of British Neurologists also advertised the study. The clinician survey was available online from Jul-Nov 22. Clinician and patient respondents in our previous studies who had given permission to be contacted for future studies were also directly emailed the survey link.

Clinicians from rheumatology, psychiatry, primary care and neurology were explicitly invited to participate. Other specialists who completed the survey were included (e.g. nephrology and immunology) but were not analysed as separate categories. Controls were recruited by asking patient respondents to forward the controls survey link to a friend with the following exclusion criteria: <18 years old, any serious physical disease such as any SARD, MS, HIV, terminal cancer. It was made clear on advertising and in the control information sheet that all types and severities of mental health and NP conditions were eligible. ‘Healthy friend’ controls are used in medical research for many reasons, particularly the convenience and greater likelihood of case-control sociodemographic similarities ^13^.

Symptoms occurring exclusively during an infection, drug or alcohol use, or attributed to a medication side-effect, were requested to be excluded from self-reports or clinician estimates.

Participant information sheets were provided online via a link at the start of each survey. Informed consent was taken by a statement at the start of the online surveys and recorded verbally on audio-recordings on the interviews

There were no pre-defined recruitment numbers for any group of participants, and interest exceeded expected quantities aside from clinicians from primary care and neurology who were challenging to recruit. The patient surveys were made available online, then each charity/support group was requested to send 2 reminders. On completion of the patient survey, the completion page contained a request to send the controls link to a friend. Patients were reminded of this link and requested to ask a friend until we had appropriate numbers of controls (n=400). Clinician recruiting continued until a minimum of 30 surveys were received from each main speciality of interest (rheumatology, psychiatry and neurology) to ensure sufficient numbers for viable statistical comparison.

1. **Data cleaning**

Data cleaning excluded patients without SARDs and those who had not completed at least 30% of the survey. Fibromyalgia as a primary disease was initially considered for inclusion due to increasing evidence of the likelihood that it is a SARD, but primary fibromyalgia participants were excluded (from this part of the study) due to not meeting the >50 Ppt threshold in total completing the survey. The disease status of controls was checked twice, once on sign up to the survey, where potential respondents with any serious physical disease were told they were ineligible. This was later checked by a question on the survey about physical and mental health conditions. All mental health conditions were eligible. Respondents with any SARD or other serious autoimmune condition were excluded (e.g. several respondents with IBD or MS) as controls. Controls with less severe and controllable common conditions such as osteoarthritis or thyroid disease were able to participate. Three clinicians completed the survey twice. Two seemingly by accident so their second attempt was deleted. One clinician requested they complete the survey again as they felt they had rushed the first completion so their first attempt was excluded.

1. **Interviews**

Interviews were carried out before, during and following the collection of quantitative data. Sampling for these participants was through collaborators’ contacts, ongoing study participants in the related LISTEN study and a selection of the most eminent UK rheumatologists. Purposive sampling from the survey results was then used to select interviewees with a range of demographic characteristics, diseases, clinician roles and opinions. Approach was by the email contact details provided on the survey. Although no-one explicitly declined to be interviewed, approx. 20% of patients and clinicians contacted did not respond to the email request for interview. Up to 2 further attempts were made before selecting another potential interviewee.

The interview used a pre-tested flexible schedule, designed to elicit and explore NP symptoms, medical experiences, the medical relationship, trust, mental health and wellbeing, concerns and views. It contained a mixture of standard questions with additional questions based on individual participant survey responses. Interviews were semi-structured, with key open-ended questions asked initially, then subsequent questions following the direction of each participant’s experiences and priorities for discussion as long as they were within the study remit.

Depending on the preference and location of the interviewee, interviews were mostly by Zoom, with some by telephone/alternative remote method. A small minority were face-to-face (n=4), and some Ppts (n=12 patients and n=8 clinicians) completed the questions (and follow up questions) by email correspondence. Email interviews were given as an option, and preferred by some participants, often if too busy or unwell for verbal interviews. They were found to produce equally rich data in our previous studies. However, in this study, particularly with patients discussing difficult NP symptoms, and clinicians discussing inter-specialism disagreements, email interviews were found to sometimes be less enlightening and contain less depth of content. Some also required several follow-up emails or a subsequent Zoom/telephone interview for more in-depth results and clarification.

Whilst we attempted to continue interviewing until saturation is reached (the point at which no novel concepts arise), we were constrained by the limited number of neurologists giving contact details and consent for interview. Their diverse and conflicting viewpoints meant that more interviews may have been revealing and attempts to interview more neurologists for INSPIRE studies are ongoing. Overall saturation in terms of clinicians and overall patients was felt to have been reached, and additional interviews were to ascertain between group differences.

1. **Analysis**

**Qualitative**

Analysis was thematic ^14^ with themes developed directly from the data. These were then tested and developed against later interviews using the constant comparative method until theoretical saturation is reached (point at which additional interviews do not provide new relevant insights or codes).

The stages of analysis involved in our qualitative research involves an inductive-deductive process:

1) Immersion in the data - Immersion in the transcripts continues throughout the whole interviewing, analysis and writing process with frequent reading of each full transcript/ communication and each combined coded component. These are viewed repeatedly both using the computer-assisted coding and by manually coding and highlighting. Ongoing stages include creating mind-maps and spreadsheets of emerging team ideas and themes. Transcripts and subsequent coded sections are read and discussed by multiple team members to improve reliability, ensure multiple perspectives and possible interpretations are represented, and individual subconscious biases are minimised.

2) Coding (classification) scheme is developed and trialled –A coding scheme is developed from initial interviews to incorporate all issues discussed. Two or more of the team do this independently, then meet to discuss. It is then tested by MS and other team members. Codes are generated directly from the interviews.

3) Coding - Each line of each interview and all qualitative responses on the survey are coded by MS using NVivo 12 software. Some sections can receive multiple codes.

4) Refining and re-coding – Double-coding of a proportion of interviews is carried out by a second (and sometimes third) co-author to ensure agreement and reliability of the coding framework. MS then presents example participant quotes and suggested coding to the wider team who give agreement or suggestions for improvements. Following discussion, the coding frame is then adapted and parts of interviews are re-coded where necessary.

5) Identification of themes - Participant extracts for each code are combined using Nvivo 12. This allows for the concurrent viewing of raw data (each transcript/emails) and the coded data. Codes are then discussed and combined into broader categories. The key themes are generated directly from the data and ensuring team discussions, including with multiple patients and clinicians. These are then refined and agreed by the team.

In addition to the standard thematic analysis detailed, this study incorporated an additional method in line with our democratic ethos and aim of co-constructing the themes between the study team and study participants. This was enabled by interviewees after the survey period being shown some provisional results/ graphs and reactions and interpretations obtained. Views of each group of interviewee (e.g. psychiatrists, rheumatologists, patients) were then discussed with subsequent interviewees from the same and other groups.

Illustrative patient quotes are used for key findings and are identifiable by participant number and other participant characteristic, such as gender, age, disease, clinician role etc. Sub/minor themes are incorporated into main themes. Opinions that deviate from the group norm are examined and discussed in detail. Participant quotes are selected to illustrate common and contrasting viewpoints. Inclusion of specific quotes does not constitute the study team’s agreement with the viewpoint, or endorsement of the scientific accuracy of the quote.

**Quantitative analysis**

Survey results are transferred directly from Qualtrics to Excel and SPSS for analysis. JB, data manager, anonymises and password protects any identifiable information.

This paper was largely qualitative, and the only quantitative data was the timings of each NP symptom which were presented as percentages of the total number of participants with SLE reporting having experienced that symptom.

1. **Research team and reflexivity**

The INSPIRE project team is multi-disciplinary and was carefully selected to encompass a broad range of expertise, perspectives and views on SARD NP symptoms, and includes: rheumatologists, psychiatrists, patients, psychologists, neurologists, a data manager and a statistician as follows:

Academic team, including behavioural and social science, methodology, qualitative, data management and statistics: James Brimicombe Rupert Harwood, Efthalia Massou, Felix Naughton, Melanie Sloan, Sam Sloan, Mary Summers, Mandeep Ubhi

Nephrology and vasculitis: David Jayne

Neurology: Guy Leschziner, Michael Zandi.

Patient collaborators and representatives: Colette Barrere, Moira Blane, Michael Bosley, Wendy Diment, Lynn Holloway, Kerryn Lyon, Kate Middleton, Ali Seamer, Denise Wheelwright

Psychiatry: James Bourgeois, Mervi Pitkanen, Thomas Pollak

Rheumatology: Laura Andreoli, Alessandra Bortoluzzi, David D’Cruz, Caroline Gordon, Chris Wincup

(Individual INSPIRE papers will be co-authored by different groupings of co-authors depending on factors including: specific interests, time, health).

Physicians and patients involved in research may have varying degrees of conscious and unconscious bias towards respective physician/patient viewpoints. The potential for any individual team member bias influencing the study was mitigated by the diverse multi-disciplinary study team providing thorough and multi-perspective ongoing feedback on the data analysis, being open about potential bias, and alert to the need to identify possible bias in themselves and each other. Patient representatives play a major role in all our studies, and were initially selected to be study team members on the basis of having a combination of positive and negative medical experiences, and all demonstrating, through their forum posts/ responses, an objectivity necessary for active involvement as collaborators in this research. Two patient team members are also ex-clinicians. Rheumatology charity staff were consulted on their opinions and added depth of understanding of these patient groups.

Interviews were carried out by MS (Behavioural science, Cambridge University), RH (PhD student and experienced social researcher, Swansea University) and MU (Psychologist, Birmingham University). All interviewers have been qualitatively trained and are experienced in interviewing. Potential bias is mitigated by all interviewers having extensive interview training and experience, and striving to maintain a neutral, objective, yet empathetic interview technique. In addition, the interview team has a broad range of socio-demographic characteristics including: an age range of 30s-50s, male and female, SE Asian and white, SARDs patients and healthy. This assists in building rapport with a large range of participants and reduces any individual bias. Interviews are transcribed verbatim and viewed/analysed by multiple members of the team. Any parts of interviews with accidentally leading questions (except for confirmatory statements to clarify the interviewer has correctly understood and interpreted the Ppts views) are deleted and not used in analysis.

A great strength of the study was the depth of knowledge of this patient group and diseases from the study clinicians, study patients and researchers. A major identified strength was that participants indicated a great deal of trust in having fellow patients interviewing and all interviewers being non-judgemental and empathetic during the interviews. This was especially important when NP symptoms can be difficult to elicit and divulge, and it seems likely that this rapport and trust contributed to the openness of interviewees and rich data provided. Clinicians surveying or interviewing their own patients is more likely to lead to social desirability bias, particularly in rheumatology where patient dependency and power differentials can be pronounced, partially due to the diagnostic difficulties and necessity for lifelong medical relationships. Patients wanting to please their own clinicians in research can significantly skew results (particularly when asking if they are satisfied with their care), and our studies therefore use non-clinician interviewers and patients from a broad range of hospitals. The lead researcher was introduced to the patient participants through the charities, forum and Facebook group moderators and answered questions regarding the study.

1. **Additional Limitations**

Although interviewees were purposively selected to ensure a wide range of demographic, disease and experience characteristics, the participants completing online surveys may not be representative of the wider rheumatology disease populations in terms of gender, ethnicity, socio-demographics and experiences. This is due to the self-selecting nature of online surveys which may also attract those with stronger opinions in both patient and clinician groups. This was mitigated somewhat (although not entirely) by phrasing of the study advertising to be neutral. Although males are in the minority in some rheumatological diseases, e.g. lupus, they are even further under-represented in online support groups and in choosing to complete online surveys. Our survey also recruited a lower proportion of patients from minority ethnic groups than is representative of the SARD population, as is common in rheumatological research. ^15, 16^ The potential lack of ethnic diversity may have influenced the results as disease severity differs between ethnic groups ^17^ and may also impact patients’ experience ^16^ and the patient-physician interaction. ^18^ However, we attempted to mitigate this by contacting all participants (who had provided contact details and permission to be contacted for interview) from non-white ethnicities and offering them an interview with MU. These are ongoing and have been amalgamated into the main research studies, and are also going to generate an additional paper about any particular challenges faced by minority groups as this is currently a very neglected area of research.

Online support groups may also attract those with more negative medical experiences in need of more information or peer support. Online recruitment may exclude patients who are more disadvantaged and unable to access/use the technology required. In terms of NP symptoms specifically, online recruitment will also likely exclude the most unwell and those currently experiencing severe NP symptoms, so our data may be somewhat skewed towards the less severe. However, it is also possible that SARDs patients in remission or with few/no current symptoms, including NP symptoms, will be less in need of support so less likely to be participating in online support groups, and therefore not have received the survey. Diagnoses were also unable to be validated, although we asked for the diagnosis/diagnoses as written on their clinic letter and excluded any participants with uncertain diagnoses. In addition, Controls may not have been representative of the general population, although common symptoms such as anxiety ^19^, and tinnitus ^20^, were similar to previously reported general population prevalences.

Clinician participants being largely recruited through twitter may not be socio-demographically representative of the whole clinician population, and those choosing to complete surveys to be of patient benefit may be those with a more patient-centred focus. For example, the extreme difference between patients feeling they rarely/ never asked about MH (>70%) and clinician respondents having much lower values of never/rarely asking (<5%) may be partially due to the clinicians who were least interested in asking patients about MH also being the least likely to respond to a survey about MH symptoms.

A significant limitation is that many (n>100) neurologists and psychiatrists withdrew from the survey at the first non socio-demographic question or during the first questions on medical relationships with rheumatology patients. As they hadn’t had the opportunity to provide contact details at this stage, we were unable to contact them to ascertain reasoning. However, unsolicited email feedback from several clinicians suggests this was largely due to these specialists having limited/no experience with rheumatology patients and thus feeling they couldn’t answer these questions with any accuracy.

Some clinician participants may have had research or clinical interests that they wished to promote through this study, either through their own interviews or by re-distributing the survey link only to those with similar research interests or views. This is likely less of a problem with rheumatologists due to greater numbers and a broad representation, but may be an issue with neurology respondents due to small numbers and strong conflicting ideology between neurologists on NP symptoms and attribution. The study team’s awareness of this potential issue helped to mitigate any undue influence, whilst also ensuring reported qualitative data is as fair and accurate representation of all Ppt views as possible. Our neurologist quantitative data in particular may not reflect the broader neurologist viewpoint.

The Covid-19 pandemic may have adversely impacted participants’ mental health, leading to higher self-reported prevalences than if the survey had been completed pre-pandemic.

This research, in combining the experiences of a range of patient and clinician perspectives, also highlighted the limitations inherent in research that presumes the views of one group of participants is the reality. It is therefore important to highlight that participants’ perceptions of the same events may differ, and triangulation of numerous viewpoints allows us to be closer to the reality without assuming that either party’s perceptions of events are superior or the ‘true’ reflection of the situation.

1. **COREQ (COnsolidated criteria for REporting Qualitative research) Checklist** ^21^

| Topic | Item No | Guide Questions/Description | Details and/or Reported on Page No. |
| --- | --- | --- | --- |
| **Domain 1: Research team and reflexivity** |  |  |  |
| *Personal characteristics* |  |  |  |
| Interviewer/facilitator | 1 | Which author/s conducted the interview or focus group? | MS, RH. MU |
| Credentials | 2 | What were the researcher’s credentials? | All qualitatively trained and experienced |
| Occupation | 3 | What was their occupation at the time of the study? | MS, MU -Research Associates. RH – PhD candidate. |
| Gender | 4 | Was the researcher male or female? | MS & MU – Female  RH - Male |
| Experience and training | 5 | What experience or training did the researcher have? | Supplementary Information |
| Relationship established | 6 | Was a relationship established prior to study commencement? | Supplementary Information |
| Participant knowledge of the interviewer | 7 | What did the participants know about the researcher? | Patient participants – all details including university and if the interviewer had a SARD.  Clinicians – professional qualifications and University. |
| Interviewer characteristics | 8 | What characteristics were reported about the interviewer/facilitator? | Age, gender, ethnicity, disease status. Supplementary Information |
| **Domain 2: Study design** |  |  |  |
| *Theoretical framework* |  |  |  |
| Methodological orientation and Theory | 9 | What methodological orientation was stated to underpin the study? | Constructionist and moderate realist |
| *Participant selection* |  |  |  |
| Sampling | 10 | How were participants selected? | Open invitation online, support groups and professional networks |
| Method of approach | 11 | How were participants approached | Twitter, disease support groups, professional networks, social media |
| Sample size | 12 | How many participants were in the study | Table 1 |
| Non-participation | 13 | How many people refused to participate or dropped out? | Supplementary Information |
| *Setting* |  |  |  |
| Setting of data collection | 14 | Where was the data collected? | Online and via various methods for interviews |
| Presence of non-participants | 15 | Was anyone else present besides the participants and researchers? | Occasional presence of family member during interviews |
| Description of sample | 16 | What are the important characteristics of the sample? | Table 1 and supplementary data 2 |
| *Data collection* |  |  |  |
| Interview guide | 17 | Were questions, prompts, guides provided by the authors? | Yes. Supplementary Information |
| Repeat interviews | 18 | Were repeat interviews carried out? | With a limited number of Pts from the LISTEN study |
| Audio/visual recording | 19 | Did the research use audio or visual recording to collect the data? | Audio. |
| Field notes | 20 | Were ﬁeld notes made during and/or after the interview or focus group? | Yes. |
| Duration | 21 | What was the duration of the inter views or focus group? | Patients – Approx 1-2.5 hours each  Clinicians - Approx 40-60 mins each. |
| Data saturation | 22 | Was data saturation discussed | Yes |
| Transcripts returned | 23 | Were transcripts returned to participants for comment and/or correction? | No, but member checking of draft manuscript occurred |
| **Domain 3: analysis and findings** |  |  |  |
| *Data analysis* |  |  |  |
| Number of data coders | 24 | How many data coders coded the data? | 2 and then a third if necessary |
| Description of the coding tree | 25 | Did authors provide a description of the coding tree? | Available on request |
| Derivation of themes | 26 | Were themes identiﬁed in advance or derived from the data? | Derived from data. |
| Software | 27 | What software, if applicable, was used to manage the data? | Nvivo 12. |
| Participant checking | 28 | Did participants provide feedback on the ﬁndings? | Yes. Member checking |
| *Reporting* |  |  |  |
| Quotations presented | 29 | Were participant quotations presented to illustrate the themes/ﬁndings? | Yes. |
| Data and findings consistent | 30 | Was there consistency between the data presented and the ﬁndings? | Yes. |
| Clarity of major themes | 31 | Were major themes clearly presented in the ﬁndings? | Yes. |
| Clarity of minor themes | 32 | Is there a description of diverse cases or discussion of minor themes? | Yes. |

**Supplementary Information References**

1. Creswell JW and Clark VLP. *Designing and conducting mixed methods research*. Sage publications, 2017.

2. Kitcher P. *Science, truth, and democracy*. Oxford University Press, 2003.

3. Schwandt TA. On Understanding Understanding. *Qualitative Inquiry* 1999; 5: 451-464. DOI: 10.1177/107780049900500401.

4. Birt L, Scott S, Cavers D, et al. Member Checking: A Tool to Enhance Trustworthiness or Merely a Nod to Validation? *Qual Health Res* 2016; 26: 1802-1811. 20160710. DOI: 10.1177/1049732316654870.

5. Christians CG. ETHICS AND POLITICS IN QUALITATIVE RESEARCH. In: 2005.

6. Sloan M, Harwood R, Gordon C, et al. Will 'the feeling of abandonment' remain? Persisting impacts of the COVID-19 pandemic on rheumatology patients and clinicians. *Rheumatology (Oxford)* 2022; 61: 3723-3736. DOI: 10.1093/rheumatology/keab937.

7. Wolcott MD and Lobczowski NG. Using cognitive interviews and think-aloud protocols to understand thought processes. *Curr Pharm Teach Learn* 2021; 13: 181-188. 20201014. DOI: 10.1016/j.cptl.2020.09.005.

8. Liang MH, Corzillius M, Bae SC, et al. The American College of Rheumatology nomenclature and case definitions for neuropsychiatric lupus syndromes. *Arthritis and rheumatism* 1999; 42: 599-608.

9. Bortoluzzi A, Scire CA, Bombardieri S, et al. Development and validation of a new algorithm for attribution of neuropsychiatric events in systemic lupus erythematosus. *Rheumatology (Oxford)* 2015; 54: 891-898. 20141021. DOI: 10.1093/rheumatology/keu384.

10. Hanly JG, Urowitz MB, Su L, et al. Short-term outcome of neuropsychiatric events in systemic lupus erythematosus upon enrollment into an international inception cohort study. *Arthritis Rheum* 2008; 59: 721-729. DOI: 10.1002/art.23566.

11. Badenoch JB, Rengasamy ER, Watson C, et al. Persistent neuropsychiatric symptoms after COVID-19: a systematic review and meta-analysis. *Brain Commun* 2022; 4: fcab297. 20211217. DOI: 10.1093/braincomms/fcab297.

12. Sloan M, Harwood R, Sutton S, et al. Medically explained symptoms: a mixed methods study of diagnostic, symptom and support experiences of patients with lupus and related systemic autoimmune diseases. *Rheumatol Adv Pract* 2020; 4: rkaa006. 20200226. DOI: 10.1093/rap/rkaa006.

13. Bunin GR, Vardhanabhuti S, Lin A, et al. Practical and analytical aspects of using friend controls in case-control studies: experience from a case-control study of childhood cancer. *Paediatr Perinat Epidemiol* 2011; 25: 402-412. 20110719. DOI: 10.1111/j.1365-3016.2011.01210.x.

14. Braun V and Clarke V. *Thematic analysis*. American Psychological Association, 2012.

15. Lima K, Phillip CR, Williams J, et al. Factors Associated With Participation in Rheumatic Disease-Related Research Among Underrepresented Populations: A Qualitative Systematic Review. *Arthritis Care Res (Hoboken)* 2020; 72: 1481-1489. DOI: 10.1002/acr.24036.

16. McNeil JN. *" I noticed something wrong": Lived experiences of women of color who faced a protracted journey to diagnosis with lupus*. Capella University, 2017.

17. Lewis MJ and Jawad AS. The effect of ethnicity and genetic ancestry on the epidemiology, clinical features and outcome of systemic lupus erythematosus. *Rheumatology (Oxford)* 2017; 56: i67-i77. DOI: 10.1093/rheumatology/kew399.

18. Chae DH, Martz CD, Fuller-Rowell TE, et al. Racial Discrimination, Disease Activity, and Organ Damage: The Black Women's Experiences Living With Lupus (BeWELL) Study. *Am J Epidemiol* 2019; 188: 1434-1443. DOI: 10.1093/aje/kwz105.

19. Löwe B, Decker O, Müller S, et al. Validation and Standardization of the Generalized Anxiety Disorder Screener (GAD-7) in the General Population. *Medical Care* 2008; 46: 266-274. DOI: 10.1097/MLR.0b013e318160d093.

20. McCormack A, Edmondson-Jones M, Somerset S, et al. A systematic review of the reporting of tinnitus prevalence and severity. *Hear Res* 2016; 337: 70-79. 20160528. DOI: 10.1016/j.heares.2016.05.009.

21. Tong A, Sainsbury P and Craig J. Consolidated criteria for reporting qualitative research (COREQ): a 32-item checklist for interviews and focus groups. *Int J Qual Health Care* 2007; 19: 349-357. 20070914. DOI: 10.1093/intqhc/mzm042.
